# Re: Pushpanathan et al.: Can OpenAI’s New o1 Model Outperform Its Predecessors in Common Eye Care Queries?

**DOI:** 10.1016/j.xops.2025.100968

**Published:** 2025-10-10

**Authors:** Yusuke Kameda, Yutaka Kaneko, Saki Takada

**Affiliations:** 1 Yotsuya-Sanchome Ekimae Eye Clinic, Tokyo, Japan; 2 Department of Ophthalmology and Visual Sciences, Yamagata University Faculty of Medicine, Yamagata, Japan

TO THE EDITOR:

We read with great interest the recent article by Pushpanathan et al^1^ evaluating the performance of OpenAI’s o1 model in ophthalmology-related queries. The study provides timely insights into the growing role of large language models in clinical practice. While we found the study informative, we would like to raise two points—one regarding methodological clarity and another suggesting a possible direction for future research informed by clinical observations.

As large language model outputs are inherently probabilistic, minor changes in parameters—particularly the temperature setting—can lead to a significant variation in responses. In the study, the default temperature range (0.7–1.0) was used without specific adjustment. By contrast, Antaki et al^2^ demonstrated improved consistency and accuracy in ophthalmic tasks when using a lower temperature (0.3). This raises an important methodological question: Did the authors observe any variability in model outputs that might have influenced the grading? We believe a discussion on this point would be helpful, especially given the growing emphasis on reproducibility in large language model–based research.^3^ If such variability was observed, it might warrant acknowledgment as a study limitation, as stated in the study by Ghanem et al.^4^

Recently, there has been an increasing number of patients using Chat Generative Pre-trained Transformer (ChatGPT) independently—likely powered by a recent model such as o1—to inquire about their symptoms and perform informal self-assessments. Anecdotally, it seems that the diagnostic accuracy of ChatGPT in such patient-led queries has improved with newer model versions. However, to our knowledge, no study has systematically evaluated ChatGPT’s diagnostic performance when used directly by patients without clinician guidance. Current studies largely examine ChatGPT’s utility from a clinician or educator perspective. Therefore, we propose that exploring this patient-facing application could provide valuable insights and would be a natural extension of this research.
